# Molecular Signaling Pathways Mediating Osteoclastogenesis Induced by Prostate Cancer Cells

**DOI:** 10.1186/1471-2407-13-605

**Published:** 2013-12-26

**Authors:** Shahrzad Rafiei, Svetlana V Komarova

**Affiliations:** 1 Department of Anatomy and Cell Biology, Faculty of Medicine, 3640 University Street, Montreal, Quebec H3A 2B2, Canada; 2 Shriners Hospital for Children-Canada, 1529 Cedar Avenue, room-300, Montreal, Quebec H3G IA6, Canada; 3 Faculty of Dentistry, McGill University, 3640 University Street, Montreal, Quebec H3A 0C7, Canada

**Keywords:** Prostate cancer, Bone metastasis, Osteoclast, Calcium signaling, NFATc1, ERK1/2

## Abstract

**Background:**

Advanced prostate cancer commonly metastasizes to bone leading to osteoblastic and osteolytic lesions. Although an osteolytic component governed by activation of bone resorbing osteoclasts is prominent in prostate cancer metastasis, the molecular mechanisms of prostate cancer-induced osteoclastogenesis are not well-understood.

**Methods:**

We studied the effect of soluble mediators released from human prostate carcinoma cells on osteoclast formation from mouse bone marrow and RAW 264.7 monocytes.

**Results:**

Soluble factors released from human prostate carcinoma cells significantly increased viability of naïve bone marrow monocytes, as well as osteoclastogenesis from precursors primed with receptor activator of nuclear factor κ-B ligand (RANKL). The prostate cancer-induced osteoclastogenesis was not mediated by RANKL as it was not inhibited by osteoprotegerin (OPG). However inhibition of TGFβ receptor I (TβRI), or macrophage-colony stimulating factor (MCSF) resulted in attenuation of prostate cancer-induced osteoclastogenesis. We characterized the signaling pathways induced in osteoclast precursors by soluble mediators released from human prostate carcinoma cells. Prostate cancer factors increased basal calcium levels and calcium fluctuations, induced nuclear localization of nuclear factor of activated t-cells (NFAT)c1, and activated prolonged phosphorylation of ERK1/2 in RANKL-primed osteoclast precursors. Inhibition of calcium signaling, NFATc1 activation, and ERK1/2 phosphorylation significantly reduced the ability of prostate cancer mediators to stimulate osteoclastogenesis.

**Conclusions:**

This study reveals the molecular mechanisms underlying the direct osteoclastogenic effect of prostate cancer derived factors, which may be beneficial in developing novel osteoclast-targeting therapeutic approaches.

## Background

Prostate cancer is estimated to be the most common cancer diagnosed in men in the United States [1], and the sixth leading cause of cancer-related deaths in affected men worldwide [2,3]. Autopsy studies have revealed that over 80% of patients with advanced prostate cancer have skeletal metastasis [4]. The growth-supportive interactions between the disseminated prostate cancer cells and bone induce heterogeneous lesions of mixed osteolytic and osteoblastic nature which disrupt bone homeostasis, leading to complications including spinal cord compression, pathological fractures, and severe bone pain [5,6]. While prostate cancer bone metastases were initially characterized to exhibit mainly osteoblastic lesions [7-10], studies have revealed the clinical importance of the lytic component of prostate cancer metastasizing to bone [11,12]. However the precise molecular basis underlying the ability of prostate cancer cells to modulate bone resorption by osteoclasts remains poorly understood.

Osteoclastogenesis is the differentiation of mono-nuclear precursors originated from hematopoietic progenitors of monocyte/macrophage lineage into mature multi-nuclear resorbing osteoclasts [13,14]. RANKL produced by cells of osteoblastic lineage plays a critical role in regulating osteoclastogenesis [15]. RANKL binds to its receptor RANK and activates a signal transduction cascade that leads to osteoclast differentiation in the presence of MCSF, the osteoclast survival factor [16]. On the other hand, osteoprotegerin (OPG) produced by osteoblasts acts as a decoy receptor for RANKL and inhibits osteoclast formation [16,17]. MCSF is also produced by osteoblasts and is critically important for survival and differentiation of osteoclasts [13,14]. TGFβ physiologically released from bone matrix also has an ability to modify osteoclast differentiation and function [18,19]. In particular, the presence of MCSF, TGFβ was shown to induce osteoclast formation from mononuclear precursors in a RANKL-independent manner [20].

When prostate cancer metastasizes to bone the normal bone homeostasis is disrupted resulting in abnormal stimulation of both osteoclastic and osteoblastic components [21]. Targeting osteoclasts is clinically beneficial for prostate cancer patients, since it has been shown that the morbidity related to skeletal events is reduced when prostate cancer patients are treated with denosumab, an inhibitor for RANKL [22,23] or zoledronic acid, an inhibitor of osteoclastic activity [24]. However, blocking RANKL does not completely block tumor development and progression in bone tissue [25]. These findings suggest that prostate cancer cells can produce other factors capable of stimulating osteoclast formation and/or function.

This study focuses on characterizing the direct osteoclastogenic effects of soluble mediators released from the prostate cancer cells, and the molecular signaling pathways induced by prostate cancer factors in osteoclast precursors. We employed conditioned medium (CM) as a source for factors produced by the human prostate carcinoma cells, PC3 and LNCaP. In vivo studies have demonstrated that following injection of PC3 or LNCaP cells in SCID mice, PC3 produces osteolytic bone metastasis, while LNCaP leads to development of osteolytic and osteoblastic bone lesions [26]. Mouse bone marrow and RAW 264.7 murine monocytic cells were used as the source of osteoclast precursors [27].

## Methods

### Cell lines and cultures

Human prostate cancer cell line, LNCaP was obtained from the American Type Culture Collection (ATCC, VA, USA; CRL-1740™) in October 2012, was expanded, frozen in aliquots in liquid nitrogen and was used within first 3 passages from originally received cells. PC3 was kindly provided by Dr. P.M. Seigel, McGill University, who received it from Dr. Mario Chevrette (McGill University). Prostate cancer cells were cultured in T-75 tissue culture flasks at 37°C in 5% CO_2_ to 80% confluence in the incubation medium RPMI-1640 (350-000-CL, Wisent Inc.) with L-glutamine and sodium bicarbonate, supplemented with 1% sodium pyruvate (600-110-EL, Wisent Inc.), 1% penicillin-streptomycin (450-201-EL, Wisent Inc.), and 10% fetal bovine serum (FBS, 080–150, Wisent Inc.). Prostate cancer incubation medium not exposed to cells was not capable to affect osteoclast formation (data not shown). Cells were rinsed with serum-free medium, and serum starved for 24 hours. CM (5.8 ± 0.6 ml/10^6^ cells) was collected, centrifuged (2000 RPM for 5 min), filtered (0.22 μm), aliquoted, and stored at -80°C until use.

RAW 264.7 mouse monocytic cell line was obtained from American Type Culture Collection, (ATCC; VA, USA, TIB-71™), cultured at a density of 15 × 10^6^ cells per T-75 tissue culture flasks in incubation medium DMEM (319-020-CL, Wisent Inc.) with 1.5 g/L sodium bicarbonate, 4.5 g/L glucose, supplemented with L-glutamine (609-065-EL,Wisent Inc.), 1% sodium pyruvate, 1% penicillin-streptomycin, and 10% FBS and was used within first 3 passages from originally received cells. To generate osteoclasts, RAW 264.7 monocytic cells were seeded at a density of 5 × 10^3^ cells/cm^2^. After 24 h, cell cultures were supplemented with RANKL (50 ng/ml) for 2 days (priming) following by application of experimental stimuli, or RANKL for additional 2 days.

Animal studies for primary osteoclast cultures were approved by the Animal Care Committee at the McGill University and conformed to the ethical guidelines of the Canadian Council on Animal Care and the Committee for Research and Ethical Issues of IASPe. Six weeks old male Balb/c mice were purchased from Charles River Co, euthanized, and their femora and tibia were dissected free of soft tissues. Bone marrow was collected from tibia and femora as previously described [28]. Cells were cultured for 24 h at a density of 15 × 10^6^ cells per T-75 tissue culture flasks in incubation medium α-MEM (12000–022, Gibco Life Tech) supplemented with 1% penicillin-streptomycin, 1% sodium pyruvate, 2.2 g/L sodium bicarbonate (USP/FCC Powder, Macron, 144-55-8), 10% FBS, 25 μg/ml MCSF (300–25, Peprotech Inc.). Non-adherent cells were collected, centrifuged, plated at a density of 7 × 10^4^ cells/cm^2^, and cultured in the presence of MCSF (50 ng/ml) and RANKL (50 ng/ml) for 3 days (priming) following by application of experimental stimuli, or RANKL for additional 2 days.

### Osteoclast identification

Osteoclast cultures were plated in 48-well plates, fixed on day 5–6 with 10% formalin (23-245-685, Fisher) for 10 min at room temperature, and stained for tartrate-resistant acid phosphatase (TRAP) by incubating for 30 min at 37°C in assay buffer (Sigma 387A). Osteoclasts were identified as TRAP-positive dark-red/purple cells with three or more nuclei. Images were recorded using a digital camera linked to PixeLINK Capture SE® Software (PixeLINK, Ottawa, Canada).

### Reagents and antibodies

Recombinant human MCSF (300–25) was from Peprotech Inc. Recombinant GST-RANKL which contains amino acids 158–316 of the mouse RANKL gene was purified from the clones kindly provided by Dr. M.F. Manolson, University of Toronto. Human recombinant OPG (500 ng/ml; amino acids 21–194 fused at the N-terminus to the Fc domain of human IgG1, ab998, Abcam) was reconstituted in PBS, aliquoted and stored at -80°C, and goat anti-human anti-MCSF blocking antibody (5 μg/ml, AB-216-NA, R&D systems) was reconstituted in PBS, aliquoted and stored at -20°C. Serum free CM of prostate cancer cells was pre-incubated with OPG and anti-MCSF for 30 and 60 min respectively, and added to the RANKL-primed precursors. TGFβ type I receptor inhibitor (5 μM, SB431542, Tocris Bioscience) was directly added to the RANKL-primed precursors for 60 min before fresh medium containing prostate cancer CM was applied. Pharmacological inhibitor of MEK, PD98059 (100 μm for RAW 264.7 and 50 μm for bone marrow cells, 513001, Calbiochem), or NFAT inhibitor 11R-VIVIT peptide (10 μm, 480401, Calbiochem) were added to RANKL-primed precursors for 1 h before application of prostate cancer CM. Calcium chelator BAPTA (50 μm, B6769, Invitrogen) was added to RANKL-primed precursors for 10 min at room temperature, then the cells were washed with PBS, and the prostate cancer CM was applied. Inhibitors were diluted in 0.1% DMSO (Sigma; D2650) which was used as a vehicle.

### Resorption assay

RAW 264.7 cells were seeded on calcium phosphate plates (3989, Corning osteo assay), cultured for 2 days with RANKL (50 ng/ml), then for 2 days with prostate cancer CM or RANKL (50 ng/ml). The images of cultures were recorded using a digital camera, and the cells were removed using 0.2% TritonX-100 in 1 M NaCl to visualize resorption pits.

### Cell viability

RAW 264.7 cells were seeded in 96-well flat-bottomed tissue culture plates (3595, Costar, Corning Inc) for 24 h, and were cultured with the indicated experimental stimuli for 2 days. 10% AlamarBlue reagent (Carlsbad, CA, USA, Invitrogen) was added to each well, and the plates were incubated for additional 20 h. Fluorescence intensity was measured using a plate reader (Infinite F200, TECAN) with filter settings of excitation 560 nm and emission 590 nm. Background reading obtained from cell culture medium with no cells or treatments was subtracted from all measurements.

### Immunoblotting

Cells were lysed in RIPA lysis buffer (50 mM Tris, pH 7.4, 150 mM NaCl, 1% Nonidet P-40, 1 mM EDTA, 1 mg/ml aprotinin, 2 mg/ml leupeptin, 0.1 mM phenylmethylsulfonyl fluoride, 20 mM sodium fluoride, 0.5 mM sodium orthovanadate), left on ice for 20 min, and centrifuged at 12,000 × g for 10 min at 4°C. Supernatant was collected, and protein content was determined using a Quant-iT™ protein assay kit (Invitrogen). Whole cell lysates (50 μg) were resolved by SDS-PAGE in 10% gel, and transferred onto a nitrocellulose transfer membranes (0.45 μm, 162–0115, Bio-Rad) using 10 mM sodium tetraborate decahydrate (1303-96-4, Fisher Scientific). The membranes were blocked with 5% non-fat dry milk for 1 h at room temperature in TBS-T buffer (10 mM Tris–HCl, pH 7.5, 150 mM NaCl, 0.05% Tween 20) followed by overnight incubation at 4°C with primary antibodies: p-ERK1/2 (1:500, 9101, Cell Signaling), ERK1/2 (1:500, 9102, Cell Signaling), or NFATc1 (1:200, 7A6, Santa Cruz Biotechnology). The blots were washed, incubated with horseradish peroxidase-conjugated secondary antibodies (anti-mouse, 170–5047; anti-rabbit, 170–5046; Bio-Rad), and visualized with a chemiluminescence system (Super signal West Pico; 34080, Pierce). Blots were re-probed with α-tubulin antibody (1:5000, T9026, Sigma-Aldrich) as a loading control. Shown are representation blots from 4 independent experiments.

### Immunofluorescence

RAW 264.7 and bone marrow cells seeded on glass cover-slips were primed with RANKL (50 ng/ml) for 2 days, and the experimental stimuli were applied for additional 2 h. Samples were fixed in 10% formalin (10 min at room temperature), washed with PBS-1X (311-13-CL, Wisent Inc.); permeabilized in 0.1% Triton X-100 diluted in PBS (10 min at room temperature), washed three times with PBS, and incubated in 1% normal goat serum (NGS) blocking buffer (in PBS, AB-108-C R & D System) overnight at 4°C. Monoclonal primary antibody to NFATc1 (1:100 in NGS, Invitrogen), was then added in blocking buffer at 4°C, for 24 h. After washing three times with PBS, the coverslips were incubated for 1 h at room temperature with the biotinylated goat anti-mouse IgG (1:200 in NGS, Invitrogen), washed three times with PBS and incubated for 1 h at room temperature with Alexa Fluor 488 conjugated streptavidin (1:100 in NGS, S11223, Invitrogen). For actin staining, osteoclast cultures were stained with Alexa Fluor® 568 phalloidin (B3475, Invitrogen) for 1 h at room temperature, washed two times with PBS. Nuclei were stained using DAPI (1:5000 in distilled water, NL5995050, Invitrogen) for 1 min followed by two washes with distilled water. Cover slips were mounted on slides using Immu-Mount (9990402, Shandon) and examined using a fluorescence inverted microscope (T2000, Nikon). For NFATc1 nuclear localization analysis, five random images per experimental condition were collected in each experiment, each image containing 32 cells ± 18 for RAW 264.7 and 4 cells ±1 for bone marrow precursors. Cells were rated positive for nuclear localization of NFATc1 if fluorescence intensity of nuclei exceeded that of the cytoplasm.

### Fluorescence measurements of cytosolic free Ca^2+^ concentration ([Ca^2+^]_i_)

RAW 264.7 cells were seeded on glass bottom 35-mm dishes culture dishes (P35G-0-4-C MatTek Corp). After 2 days priming with 50 ng/ml RANKL, cells were washed twice with DMEM containing 10 mM HEPES (330-050-EL, Wisent Inc.), and incubated in dark with 1.5 μM fura-2-AM (F1221, Invitrogen) for 40 min, at room temperature. Cultures were washed, and fresh DMEM with 10 mM HEPES, containing no additions, RANKL (50 ng/ml) or 10% prostate cancer CM were applied for 15 min, after which changes in calcium levels were recorded for 120 s.

### Statistical analyses

Data were presented as means ± standard error of the mean (SEM), sample size (n) indicates the number of independent experiments. Differences were assessed by Student’s *t-*test or ANOVA for multiple group comparisons, and accepted as statistically significant at p < 0.05.

## Results

### Soluble factors produced by prostate cancer cells do not induce osteoclast formation from naïve monocytes, but increased their viability

**Figure 1:**
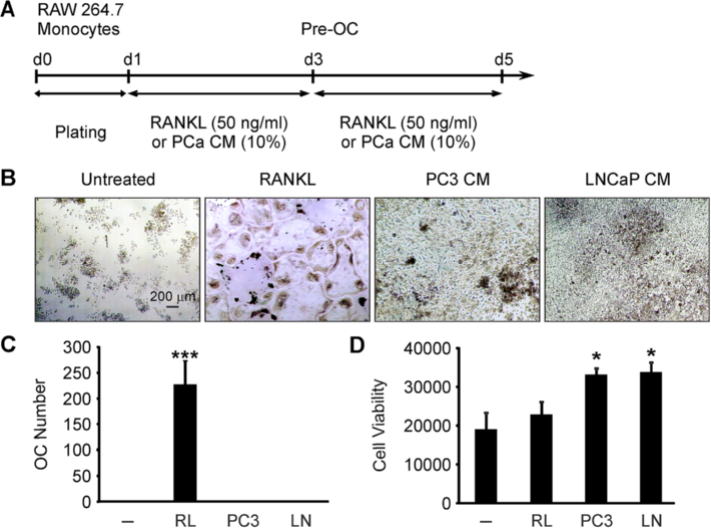
**Soluble factors produced by prostate cancer cells do not induce osteoclast formation from naïve RAW 264.7 precursors but increase the cell viability.** RAW 264.7 monocytic cells were cultured for 4 days untreated (*negative control*), treated with 50 ng/ml RANKL (RL*, positive control*), or supplemented with 10% serum free CM from PC3 or LNCaP, fixed, and stained for TRAP. **A)** Schematic overview of experimental setup for osteoclastogenesis assay from RAW 264.7 cells. **B)** Representative images of cultures after 4 days of treatment. **C)** Average osteoclast numbers formed in different cultures. Data are means ± SEM; n = 10 independent experiments; ***P < 0.001 indicates significance compared to untreated cells as assessed by Student’s *t-*test. **D)** RAW 264.7 cells were cultured for 2 days untreated, treated with 50 ng/ml RANKL, or supplemented with 10% PC3 or LNCaP CM. The cells were then incubated with AlamarBlue reagent for 20 h, and cell viability was assessed as the average fluorescence intensity of viable cells subtracted from the readings obtained from the medium. Data are means ± SEM; n = 5 experiments; *P < 0.05 indicate significance compared to negative control as assessed by Student’s *t-*test.

It was previously shown that prostate cancer cells produce factors that directly stimulate osteoclast formation from naïve monocytes [5,29]. We cultured RAW 264.7 monocytes for 4 days untreated as negative control, treated with RANKL (50 ng/ml) as positive control, or supplemented with 10% serum free CM of prostate cancer cells, PC3 or LNCaP (Figure 1A). In negative control cultures, RAW 264.7 cells formed only monocytic colonies. In positive control cultures, large multinucleated osteoclasts were observed (Figure 1B, C). Prostate cancer CM did not induce osteoclast formation from naïve RAW 264.7 cells (Figure 1B, C), however, the precursor cell density was visibly affected (Figure 1B). We directly assessed cell viability of untreated, RANKL- or prostate cancer CM-treated precursors, and have found that soluble factors secreted by prostate cancer cells enhanced monocyte viability (Figure 1D).

### Soluble factors produced by prostate cancer cells induce osteoclast formation from RANKL-primed osteoclast precursors

**Figure 2:**
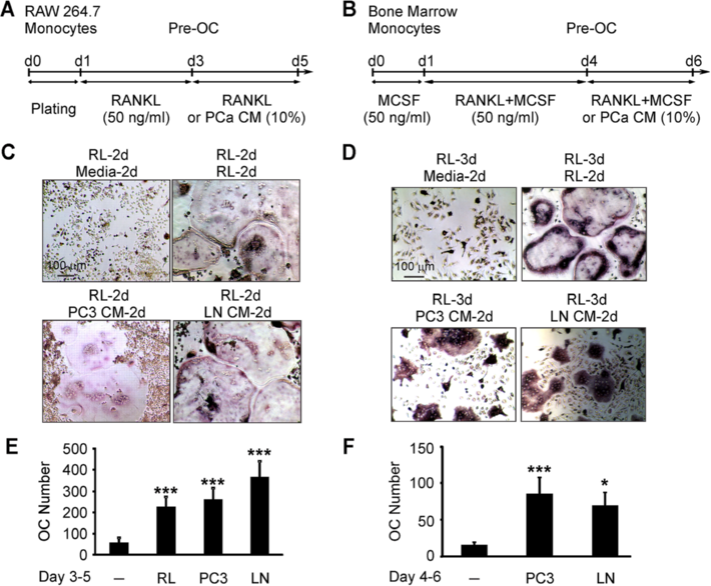
**Soluble factors produced by prostate cancer cells increase osteoclast formation from RANKL-primed precursors. A, B)** Schematic overviews of experimental setup for osteoclastogenesis assay, from **(A)** RAW 264.7 and **(B)** bone marrow cells. RAW 264.7 monocytes were first primed with 50 ng/ml RANKL (RL) for 2 days, and bone marrow cells for 3 days; then the osteoclast precursors (Pre-OC) were cultured untreated (*negative control*), treated with RANKL (*positive control*), or supplemented with 10% PC3 or LNCaP CM. **C, D)** Representative images of osteoclasts formed in different conditions from RAW 264.7 **(C)**, and bone marrow **(D)** cells. **E, F)** Average osteoclast numbers formed in different conditions from RAW 264.7 **(E)** and bone marrow **(F)** cells. Data are means ± SEM; n = 10 experiments for RAW 264.7 cells, n = 7 for bone marrow cells; *P < 0.05, ***P < 0.001 indicate significance compared to negative control as assessed by Student’s *t-test*.

We next assessed if factors secreted by prostate cancer cells can augment osteoclast formation from RANKL-primed osteoclast precursors. RAW 264.7 or bone marrow cells were treated with RANKL for a short period of time: 2 days for RAW 264.7 or 3 days for bone marrow cells (we have found in preliminary experiments that bone marrow cells require longer priming, suggesting that they are less differentiated compared to RAW264.7 cells). Then, the cells were cultured for additional 2 days untreated (*negative control*), continuously treated with RANKL (50 ng/ml, *positive control*) or exposed to 10% of PC3 or LNCaP CM (Figure 2A, B). In negative control cultures, only osteoclast precursors and a few small osteoclasts were formed. In positive control cultures, large multinucleated osteoclasts were observed. Importantly, priming with RANKL resulted in developing precursor sensitivity to soluble factors produced by prostate cancer cells, evident in a significant increase in numbers of large multinucleated osteoclasts in PC3 and LNCaP CM-treated cultures (Figure 2C-F).

**Figure 3:**
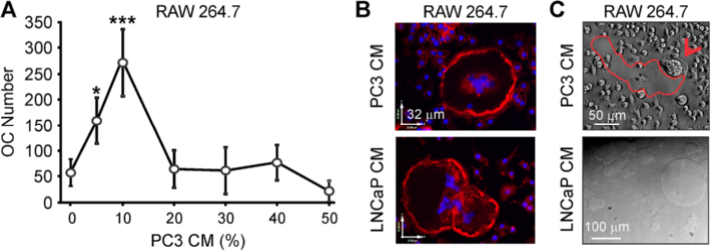
**Osteoclasts are induced by prostate cancer CM in a concentration-dependent manner, exhibit characteristic actin structure, and resorb calcified matrices. A)** RAW 264.7 cells were first primed with 50 ng/ml RANKL for 2 days, then cultured for additional 2 days untreated (*negative control*), or exposed to different dilutions of PC3 CM , and the average number of osteoclasts was assessed. Data are means ± SEM; n = 3-10 experiments; *P < 0.05, ***P < 0.001 indicate significance compared to negative control as assessed by ANOVA following by Tukey posttest. **B)** RANKL-primed osteoclast precursors supplemented for 2 days with 10% CM of PC3 (*top*) or LNCaP (*bottom*) were fixed, actin filaments were labeled using Alexa Fluor® 568 phalloidin (*red*), and nuclei were counterstained with DAPI (*blue*). Representative images of actin rings in osteoclasts formed after exposure to prostate cancer CM. **C)** RAW 264.7 cells were placed on calcium phosphate substrates, primed with RANKL for 2 days, and exposed to 10% PC3 (*top*) or LNCaP (*bottom*) CM for 2 days. Top: Representative image of active multinucleated osteoclast (*red arrow*) forming a resorption pit (*red outline*). Bottom: Representative image of resorbed areas after osteoclasts were removed.

We investigated the concentration-dependence of the osteoclastogenic effect of the PC3 CM using different dilutions (5-50%) and found that when RANKL-primed precursor cultures were supplemented with 5-10% PC3 CM, osteoclast number was significantly increased. Further increase in the PC3 CM from 10 to 50% resulted in decline in osteoclastogenic efficiency, possibly reflecting depletion of nutrients in the medium due to conditioning by the PC3 cells (Figure 3A). Osteoclasts induced by prostate cancer CM exhibited characteristic features of functional resorptive cells such as actin rings associated with resorption (Figure 3B), and were capable of resorbing mineralized matrices (Figure 3C).

### Osteoclastogenesis induced by soluble factors produced by prostate cancer cells is not mediated by RANKL

**Figure 4:**
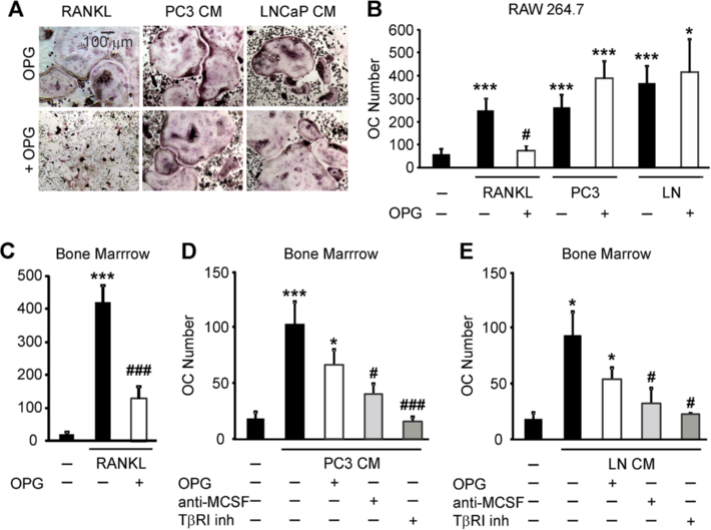
**Soluble factors produced by prostate cancer cells increase osteoclast formation in RANKL-independent manner. A, B)** RAW 264.7 cells were primed with RANKL for 2 days, then cultured for 2 days untreated (*negative control*), with RANKL (*positive control*) or exposed to 10% PC3 or LNCaP CM, in the absence (*black bars*) or presence of 500 ng/ml OPG (*white bars*), and the average number of osteoclasts was assessed. **A)** Representative images of osteoclasts induced by RANKL or prostate cancer CM in the absence (*top*), or presence (*bottom*) of OPG. **B)** Average number of osteoclasts formed in different conditions. Data are means ± SEM; n = 4-10 experiments, except for RANKL and OPG, where n = 2 repeats. **C-E)** Bone marrow cells were primed with RANKL for 3 days, and cultured for 2 days untreated *(negative control)*, treated with RANKL (*positive control*) **(C)** or exposed to 10% CM of PC3 **(D)** or LNCaP **(E)** cells, in the absence (*black bars*), or presence of 500 ng/ml OPG (*white bars*), or 5 μg/ml anti-MCSF blocking antibody (*light gray bars*) or TβRI inhibitor (5 μM, *dark gray bars*) and the average osteoclast numbers were assessed. OPG and anti-MCSF were added to prostate cancer CM for 30–60 min prior to addition to osteoclast precursors, TβRI inhibitor was added to the osteoclast precursor cultures for 60 min prior to addition of prostate cancer CM. Data are means ± SEM; n = 3-7 experiments. *P < 0.05, ***P < 0.001 indicate significance compared to negative control; ^#^P < 0.05, ^###^P < 0.005 indicate significance as compared to no inhibitor as assessed by Student’s *t-test*, no significant difference between samples treated with CM with and without OPG.

We investigated if the effects of prostate cancer CM may be mediated by RANKL produced by prostate cancer cells. We pre-incubated prostate cancer CM with RANKL decoy receptor OPG (500 ng/ml), and then added to the RANKL-primed precursors. OPG did not attenuate osteoclastogenic effect of PC3 or LNCaP CM in RANKL-primed RAW 264.7 (Figure 4A and B), or bone marrow cells (Figure 4D and E). At the same time, when added at the same concentration OPG dramatically inhibited RANKL-induced osteoclastogenesis (Figure 4). These data indicate that soluble factors produced by prostate cancer cells induce osteoclast formation in RANKL independent manner.

We next assessed if anti-MCSF blocking antibody will affect the action of prostate cancer on osteoclast formation. Prostate cancer CM was pre-incubated with anti-MCSF blocking antibody (5 μg/ml) and then added to the RANKL-primed precursors from bone marrow. We have found that blocking MCSF significantly attenuated the effect of prostate cancer CM on osteoclastogenesis (Figure 4D and E).

We examined the involvement of TβRI in prostate cancer induced osteoclastogenesis, by using pharmacological inhibitor of TβRI kinase inhibitor. RANKL-primed bone marrow precursors were cultured with prostate cancer CM in presence and absence of TβRI kinase inhibitor or vehicle (DMSO, 0.1%). Inhibition of TβRI significantly decreased prostate cancer CM-induced osteoclast formation from RANKL-primed precursors (Figure 4D and E).

### Soluble factors produced by prostate cancer cells induce calcium/NFATc1 signaling in osteoclast precursors

**Figure 5:**
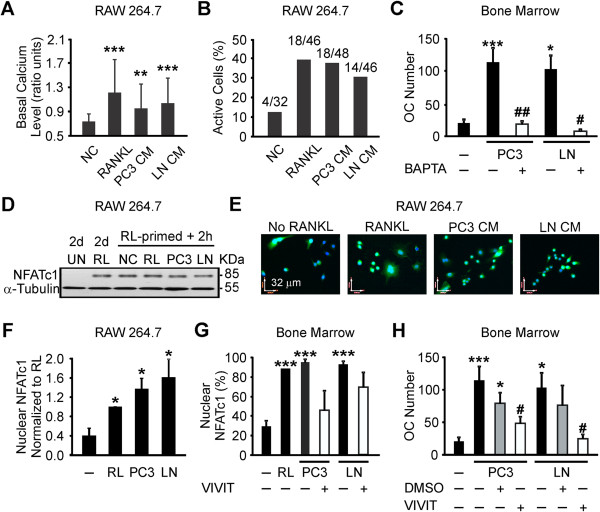
**Prostate cancer-derived factors induce calcium/NFATc1 signaling in osteoclast precursors. A, B)** RANKL-primed RAW 264.7 cells were loaded with fura-2-AM, cultured for 15 min untreated (*negative control*), with 50 ng/ml RANKL, or 10% PC3 or LNCaP CM, and imaged for 2 min. **A)** Average basal calcium levels. Data are means ± SD; n = 32-48 cells per condition; *significance compared to negative control as assessed by Student’s *t-test*. **B)** Percentage of active cells classified as having standard deviation of basal calcium above 0.05. **C)** RANKL-primed bone marrow precursors were pre-treated with 50 μM BAPTA (*white bars*) and exposed to 10% PC3 or LNCaP CM for 2 days, and average number of osteoclasts was assessed. Data are means ± SEM; n = 3-7 experiments; *significance compared to negative control; ^#^significance compared to no inhibitor. **D, F)** Protein was extracted or samples were fixed from untreated or RANKL-primed RAW 264.7 cells, or RANKL-primed precursors cultured for 2 h untreated, with RANKL, or with 10% PC3 or LNCaP CM. **D)** NFATc1 protein levels, α-tubulin as a loading control. **E)** NFATc1 localization (*green*); nuclei were counterstained using DAPI (*blue*). **F)** Average NFATc1 nuclear localization normalized to the continuous RANKL (RL) treatment. Data are means ± SEM; n = 3 experiments. **G, H)** RANKL-primed bone marrow cells were pretreated with 0.1% DMSO vehicle (*grey bars*), or 50 μM NFAT inhibitor VIVIT (*white bars*), and cultured untreated, treated with RANKL, or with 10% PC3 or LNCaP CM for 2 h to examine NFATc1 localization; or 2 days to assess osteoclast formation. **G)** Average percentage of cells with nuclear NFATc1. Data are means ± SEM; n = 3 experiments. **H)** Average number of osteoclasts. Data are means ± SEM; n = 3-7 experiments; *significance compared to negative control, ^#^significance as compared to no inhibitor.

Calcium signaling has been shown to be critical for both RANKL [30], and breast cancer factors-induced osteoclastogenesis from RANKL-primed osteoclast precursors [28,31]. RANKL-primed RAW 264.7 cells were loaded with a calcium-sensitive dye fura-2-AM, washed and incubated for 15 min in fresh media containing no additions, RANKL (50 ng/ml), or 10% prostate cancer CM. Changes in cytosolic free calcium concentration ([Ca^2+^]_i_) were recorded for 120 s. We assessed average basal calcium levels over 120 s, and fluctuations in basal levels (known to be important for osteoclastogenesis) as standard deviation of basal levels. The precursor was considered to be “active” if the standard deviation exceeded that of 0.05 ratio units. We have found that addition of RANKL or 10% of PC3 or LNCaP CM to RANKL-primed precursors significantly increased average basal calcium level (Figure 5A), as well as the percentage of active cells in the population (Figure 5B). To assess if calcium signaling is important for osteoclastogenesis induced by prostate cancer CM, we pretreated RANKL-primed bone marrow precursors with vehicle (DMSO) or calcium chelator BAPTA for 10 min, washed and supplemented with 10% prostate cancer CM for 2 days. Inhibition of calcium signaling using BAPTA significantly impaired the ability of PC3 or LNCaP CM to induce osteoclast formation (Figure 5C).

**Figure 6:**
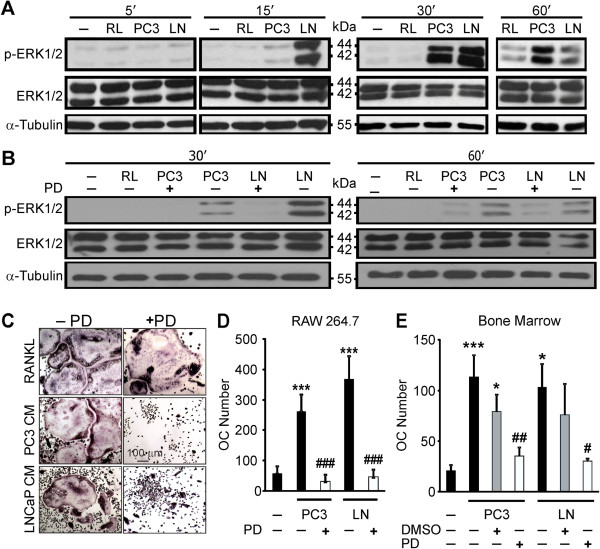
**Prostate cancer-derived factors induce activation of MEK/ERK pathway in osteoclast precursors. A)** RANKL-primed RAW 264.7 cells were cultured for 5–60 min untreated (*negative control*), treated with RANKL, or exposed to 10% PC3 or LNCaP CM. Total protein was extracted and the levels of phosphorylated ERK1/2 (p-ERK1/2), total ERK1/2, and α-tubulin were assessed by immunoblotting. **B)** RANKL-primed RAW 264.7 cells were pretreated for 1 h with 0.1% DMSO vehicle, or 100 μM MEK inhibitor (PD98059), washed and cultured for 30–60 min untreated, treated with RANKL, or 10% PC3 or LNCaP CM. Total protein was extracted and immunoblotted for p-ERK1/2, total ERK1/2 and α-tubulin. **C-E)** RANKL-primed RAW 264.7 **(C-D)**, or bone marrow **(E)** cells were pretreated for 1 h with 0.1%, DMSO vehicle (*gray bars*), or MEK inhibitor, 100 μM for RAW 264.7 and 50 μM for bone marrow cells (*white bar*), washed and cultured for 2 days (*negative control*), treated with RANKL, or 10% PC3 or LNCaP (*right column*), and the average osteoclast numbers were assessed. **C)** Representative images of osteoclasts formed from RAW 264.7 cells in different conditions. **D-E)** Average number of osteoclasts formed in RAW 264.7 **(D)** or bone marrow **(E)** cultures at different conditions. Data are means ± SEM; n = 4-10 experiments for RAW 264.7 cells, n = 3-7 experiments for bone marrow cells; *P < 0.05, ***P < 0.001 indicate significance compared to negative control; ^#^P < 0.05, ^##^P < 0.01, ^###^P < 0.001 compared to no inhibitor, assessed by Student’s *t-test*.

Since NFATc1 is a calcium-dependent osteoclastogenic transcription factor, highly up-regulated during osteoclast formation [30,32], and involved in breast cancer-induced osteoclastogenesis [33]; we next examined if NFATc1 mediates the osteoclastogenic effects of prostate cancer CM. We investigated the effect of prostate cancer CM on NFATc1 protein expression levels and cellular localization in RANKL-primed precursors exposed to prostate cancer CM for 2 h. While priming with RANKL resulted in significant increase in NFATc1 protein levels, no additional effect of prostate cancer CM was observed (Figure 5D). Using immunofluorescence, we assessed NFATc1 localization. When RANKL-primed precursors were cultured for 2 h without RANKL, only 22-30% of precursors exhibited nuclear localization of NFATc1 (Figure 6E-G). In contrast, 42-90% of osteoclast precursors exhibited nuclear NFATc1 in cultures continuously treated with RANKL. Exposure of RANKL-primed precursors to 10% prostate cancer CM resulted in significant increase in the percentage of precursors (69-97% for PC3, 80-93% for LNCaP) exhibiting nuclear NFATc1 compared to negative control (Figure 6E-G).

To further confirm that the effect of prostate cancer CM on osteoclastogenesis is mediated by NFATc1 nuclear translocation, we pretreated RANKL-primed bone marrow precursors for 1 h with vehicle (DMSO) or NFAT inhibitor, VIVIT. Prostate cancer CM-induced NFATc1 nuclear translocation was attenuated by VIVIT (Figure 6G). Osteoclast formation induced by prostate cancer CM was significantly reduced in RANKL-primed bone marrow precursors exposed to VIVIT compared to control (Figure 6H). Thus, prostate cancer-derived factors can substitute for RANKL in maintaining calcium signaling and NFATc1 activity.

### Soluble factors produced by prostate cancer cells induce osteoclastogenesis through activation of MEK/ERK signaling pathway

ERK activation induced by RANKL is known to be involved in osteoclastogenesis [34]. To investigate if ERK activation is involved in prostate cancer CM-induced osteoclastogenesis, we cultured RANKL-primed RAW 264.7 osteoclast precursors untreated, treated with RANKL (50 ng/ml), or supplemented with 10% PC3 or LNCaP CM for 5–60 min. Whole cell extracts were collected and ERK1/2 phosphorylation was assessed using immunoblotting against p-ERK1/2 (Figure 6A, *top row*). Total ERK1/2 (Figure 6A, *middle row*) and α-tubulin (Figure 6A, *bottom row*) were used as internal and loading controls respectively. Prostate cancer CM induced prolonged ERK1/2 phosphorylation that became evident at 15 min, reached maximum at 30 min, and was maintained after 60 min. ERK1/2 total levels were not affected by the treatments. Pretreatment of RANKL-primed RAW 264.7 precursors with pharmacological inhibitor of MEK1/2, PD98059 attenuated ERK1/2 activation both at 30 and 60 min after exposure to prostate cancer CM (Figure 6B). Inhibition of ERK1/2 phosphorylation significantly impaired prostate cancer CM-induced osteoclastogenesis from RAW 264.7 and bone marrow precursors (Figure 6C-E). In contrast, MEK1/2 inhibitor had only small impact on RANKL induced osteoclast formation (Figure 6C). These data suggest that soluble factors produced by prostate cancer cells induce osteoclastogenesis through activation of MEK/ERK pathway.

## Discussion

This study reports that soluble factors produced by prostate cancer cells directly induced osteoclast formation from precursors primed with RANKL for a short period of time. In contrast, prostate cancer-derived factors were not capable of inducing osteoclast formation from naïve precursors. We have found that while RANKL was important to convey sensitivity to cancer derived factors for osteoclast precursors, the subsequent osteoclast formation was not mediated by RANKL. Our data demonstrate that soluble factors produced by prostate cancer cells induce osteoclast formation through activation of calcium/NFATc1 and MEK/ERK signaling pathways.

Previous studies have revealed that factors produced by prostate cancer cells directly induce osteoclastogenesis in both RANKL-dependent [21,29], and RANKL-independent manner [5,29]. While prostate cancer cells have been shown to produce soluble RANKL [21,35], the amount was measured to be 10-fold lower than the levels produced by osteoblasts [5]. It is difficult to directly compare the results obtained in different studies, because different osteoclastogenic assays as well as conditioned medium preparations were used. In addition, it has been now recognized that cell lines, including prostate cancer and monocytic cells exhibit significant heterogeneity [36-39]. The main difference between our study and the previous ones is that we did not observe osteoclastogenesis when prostate cancer CM was applied to naïve osteoclast precursors. In contrast, we have found that cell viability of precursors was significantly improved in the presence of prostate cancer factors, which could potentially contribute to increased osteoclastogenesis in different osteoclastogenesis assay. In our study, prostate cancer factors were not able to induce osteoclastogenesis unless monocyte precursors were first primed with RANKL for 2–3 days. These data are similar to the effects of breast cancer cells on osteoclast formation [31,33,40], which were also found to occur in a RANKL-independent manner. Thus, our study suggests that RANKL is important in cancer-induced osteoclastogenesis for the initial priming of osteoclast precursors; however, in the later stages osteoclastogenesis can proceed without RANKL, providing an explanation for the lack of complete inhibition of osteoclast numbers after blocking RANKL signaling [41].

Exposure to prostate cancer factors results in formation of functional osteoclasts, evident by the presence of large osteoclast actin rings that are indicative of formation of sealing zones, a unique cell adhesion structures established at sites of osteoclast attachment to the bone surface [42,43]. Importantly, osteoclasts formed in the presence of prostate cancer cells were capable of resorbing mineralized matrices. We observed that only 5 to 10% dilutions of prostate cancer CM were capable to induce osteoclastogenesis from RANKL-primed RAW 264.7 precursors, while further increase in the amount of prostate cancer CM (20 to 50%) resulted in blunting the osteoclastogenic effects of CM. This may be consequent to the depletion of nutrients in prostate cancer CM, or to the presence of different active ingredients with competing actions.

We have demonstrated that prostate cancer factors induce osteoclastogenesis from late precursors in a RANKL-independent manner. Inhibition of TGFβ signaling in osteoclast precursors or depletion of MCSF in prostate cancer CM significantly attenuated osteoclastogenesis. TGFβ signaling has a key role in enhancing cancer progression and cancer induced bone metastasis [33,44]. Inhibition of TGFβ signaling in the mouse model of osteoblastic bone metastasis resulted in significant decrease in tumor incidence [45], however it was mostly attributed to the effects of TGFβ on osteoblasts. Importantly, PC3 and LNCaP prostate cancer cells has been shown to produce very low amounts of TGFβ [46,47], 10-100 times less than TGFβ levels reported in the fetal bovine serum by Thermo Scietific (http://www.thermoscientific.com) in December 2013, leading to the suggestion that in vitro cancer cells are more likely to act through activating TGFβ present in serum [46]. MCSF was reported to promote mature osteoclast survival and motility [48], and recently activation of mature osteoclasts, bone resorption [49]. Thus, our data suggest that TGFβ and MCSF may synergize with other soluble factors produced by prostate cancer in inducing osteoclastogenesis.

To characterize the signaling pathways induced in osteoclast precursors by prostate cancer cells, we first examined calcium/NFATc1 signaling. It has been well documented that RANKL stimulates calcium oscillations, resulting in sustained activation and up regulation of NFATc1 required for osteoclast differentiation [30,32]. In addition, we have previously shown that breast cancer cells produce factors capable of inducing calcium signaling and maintaining NFATc1 activation in RANKL-primed osteoclast precursors [28,31,33]. In this study, we demonstrated that soluble factors produced by prostate cancer increase basal calcium as well as the proportion of cells with active fluctuations in calcium levels in RANKL-primed osteoclast precursors. Moreover, blocking changes in [Ca^2+^]_i_ using intracellular chelator BAPTA prevented the osteoclastogenic effects of prostate cancer factors. RANKL is known to strongly up-regulate protein expression of NFATc1, which was recognized as an essential osteoclastogenic transcription factor [30]. Inactive NFATc1 is maintained in the cytosol in a hyper-phosphorylated form. Activation and nuclear translocation of NFATc1 requires stimulation of phosphatase calcineurin, which is in turn activated by calcium signaling [30,50]. We have found that in RANKL-primed precursors NFATc1 protein levels were significantly increased compared to naïve precursors, and were not affected by exposure to prostate cancer CM. In contrast, nuclear localization of NFATc1 was highly sensitive to the presence of RANKL, and was effectively maintained by prostate cancer factors. Inhibition of NFATc1 using cell-permeable peptide inhibitor VIVIT significantly interfered with the ability of prostate cancer-derived factors to induce osteoclastogenesis. Thus, prostate cancer factors were found to induce calcium signaling supporting NFATc1 activation in RANKL-primed osteoclast precursors. It is likely that induction of NFATc1 expression that occurred during priming of osteoclast precursors with RANKL was necessary for acquisition of their sensitivity to prostate cancer factors.

In addition to the calcium/NFATc1 signaling pathways, we have demonstrated that soluble factors produced by prostate cancer cells also promoted ERK1/2 activation. We have found that prostate cancer factors induce prolonged phosphorylation of ERK1/2, which was abolished by MEK1/2 inhibitor PD98059. Importantly, osteoclastogenesis induced by prostate cancer factors was drastically reduced when MEK/ERK activation was prevented by PD98059. MAP kinases have been previously shown to play an important role in osteoclast formation and functions [34,51]. However, in our study inhibition of ERK1/2 had only minor effect on RANKL-induced osteoclastogenesis, which is consistent with published findings [50]. While we have previously shown that breast cancer factors also induce ERK1/2 phosphorylation acting through TGFβ-dependent and independent mechanisms, inhibition of MEK was not effective in preventing breast cancer factors-induced osteoclastogenesis [31]. Thus, activation of MEK/ERK signaling pathway exhibited features unique to the osteoclastogenic effects of soluble factors produced by prostate cancer cells.

## Conclusions

This study reveals the molecular mechanisms underlying the direct osteoclastogenic effect of prostate cancer derived factors on osteoclast precursors. Although strong osteoclast targeting therapies, including bisphosphonates [24] and RANKL-targeting denosumab [22,23] are already used to treat patients with bone metastases originating from prostate cancer, drug resistance or intolerance compels the continued search of new treatments. Since both breast and prostate cancer patients suffer from frequent bone metastases, it is important to understand potential similarities and differences in the interactions of breast and prostate cancer cells with bone microenvironment. We have found that many prostate cancer-induced osteoclast signaling pathways were similar to those induced by breast cancer factors, supporting the notion that specific targeting of osteoclastogenic signaling can be effective to treat both breast and prostate cancer metastasis to bone, even if the mediators produced by these cancers are different. In addition, we have identified ERK1/2 as a unique target employed by prostate cancer cells to induce osteoclastogenesis.

## Competing interests

The authors declare that they have no competing interests.

## Authors’ contributions

SR contributed to experimental design, analysis and interpretation of data, and performed the experiments. SVK contributed to experimental design, analysis and interpretation of data. Both authors have been involved in drafting and revising the manuscript and have given final approval of the version to be published.

## Pre-publication history

The pre-publication history for this paper can be accessed here:

http://www.biomedcentral.com/1471-2407/13/605/prepub

## References

[B1] Siegel R Naishadham D Jemal A Cancer statistics, 2013 CA Cancer J Clin 2013 13 1 11 30 10.3322/caac.21166 23335087

[B2] Thobe MN Clark RJ Bainer RO Prasad SM Rinker-Schaeffer CW From prostate to bone: key players in prostate cancer bone metastasis Cancers (Basel) 2011 13 1 478 493 21603150 10.3390/cancers3010478 PMC3096870

[B3] Jemal A Bray F Center MM Ferlay J Ward E Forman D Global cancer statistics CA Cancer J Clin 2011 13 2 69 90 10.3322/caac.20107 21296855

[B4] Bubendorf L Schopfer A Wagner U Sauter G Moch H Willi N Gasser TC Mihatsch MJ Metastatic patterns of prostate cancer: an autopsy study of 1,589 patients Hum Pathol 2000 13 5 578 583 10.1053/hp.2000.6698 10836297

[B5] Lu Y Cai Z Xiao G Keller ET Mizokami A Yao Z Roodman GD Zhang J Monocyte chemotactic protein-1 mediates prostate cancer-induced bone resorption Cancer Res 2007 13 8 3646 3653 10.1158/0008-5472.CAN-06-1210 17440076

[B6] Coleman RE Skeletal complications of malignancy Cancer 1997 13 8 Suppl 1588 1594 9362426 10.1002/(sici)1097-0142(19971015)80:8+<1588::aid-cncr9>3.3.co;2-z

[B7] Mundy GR Mechanisms of osteolytic bone destruction Bone 1991 13 Suppl 1 S1 S6 1954046 10.1016/8756-3282(91)90057-p

[B8] Berruti A Piovesan A Torta M Raucci CA Gorzegno G Paccotti P Dogliotti L Angeli A Biochemical evaluation of bone turnover in cancer patients with bone metastases: relationship with radiograph appearances and disease extension Br J Cancer 1996 13 12 1581 1587 10.1038/bjc.1996.298 8664134 PMC2074561

[B9] Jung K Lein M Stephan C von Hosslin K Semjonow A Sinha P Loening SA Schnorr D Comparison of 10 serum bone turnover markers in prostate carcinoma patients with bone metastatic spread: diagnostic and prognostic implications Int J Cancer 2004 13 5 783 791 10.1002/ijc.20314 15252851

[B10] Charhon SA Chapuy MC Delvin EE Valentin-Opran A Edouard CM Meunier PJ Histomorphometric analysis of sclerotic bone metastases from prostatic carcinoma special reference to osteomalacia Cancer 1983 13 5 918 924 10.1002/1097-0142(19830301)51:5<918::AID-CNCR2820510526>3.0.CO;2-J 6681595

[B11] Clarke NW McClure J George NJ Morphometric evidence for bone resorption and replacement in prostate cancer Br J Urol 1991 13 1 74 80 10.1111/j.1464-410X.1991.tb15260.x 1873694

[B12] Corey E Brown LG Kiefer JA Quinn JE Pitts TE Blair JM Vessella RL Osteoprotegerin in prostate cancer bone metastasis Cancer Res 2005 13 5 1710 1718 10.1158/0008-5472.CAN-04-2033 15753366

[B13] Xing L Xiu Y Boyce BF Osteoclast fusion and regulation by RANKL-dependent and independent factors World J Orthop 2012 13 12 212 222 10.5312/wjo.v3.i12.212 23362465 PMC3557323

[B14] Boyce BF Advances in osteoclast biology reveal potential new drug targets and new roles for osteoclasts J Bone Miner Res 2013 13 4 711 722 10.1002/jbmr.1885 23436579 PMC3613781

[B15] Jimi E Akiyama S Tsurukai T Okahashi N Kobayashi K Udagawa N Nishihara T Takahashi N Suda T Osteoclast differentiation factor acts as a multifunctional regulator in murine osteoclast differentiation and function J Immunol 1999 13 1 434 442 10384146

[B16] Lacey DL Timms E Tan HL Kelley MJ Dunstan CR Burgess T Elliott R Colombero A Elliott G Scully S Osteoprotegerin ligand is a cytokine that regulates osteoclast differentiation and activation Cell 1998 13 2 165 176 10.1016/S0092-8674(00)81569-X 9568710

[B17] Nakashima T Hayashi M Takayanagi H New insights into osteoclastogenic signaling mechanisms Trends Endocrinol Metab 2012 13 11 582 590 10.1016/j.tem.2012.05.005 22705116

[B18] Centrella M Horowitz MC Wozney JM McCarthy TL Transforming growth factor-beta gene family members and bone Endocr Rev 1994 13 1 27 39 8156937 10.1210/edrv-15-1-27

[B19] Zhou H Choong PC Chou ST Kartsogiannis V Martin TJ Ng KW Transforming growth factor beta 1 stimulates bone formation and resorption in an in-vivo model in rabbits Bone 1995 13 4 Suppl 443S 448S 8579950 10.1016/8756-3282(95)00324-7

[B20] Itonaga I Sabokbar A Sun SG Kudo O Danks L Ferguson D Fujikawa Y Athanasou NA Transforming growth factor-beta induces osteoclast formation in the absence of RANKL Bone 2004 13 1 57 64 10.1016/j.bone.2003.08.008 14751563

[B21] Zhang J Dai J Qi Y Lin DL Smith P Strayhorn C Mizokami A Fu Z Westman J Keller ET Osteoprotegerin inhibits prostate cancer-induced osteoclastogenesis and prevents prostate tumor growth in the bone J Clin Invest 2001 13 10 1235 1244 10.1172/JCI11685 11375413 PMC209296

[B22] Smith MR Egerdie B Hernandez Toriz N Feldman R Tammela TL Saad F Heracek J Szwedowski M Ke C Kupic A Denosumab in men receiving androgen-deprivation therapy for prostate cancer N Engl J Med 2009 13 8 745 755 10.1056/NEJMoa0809003 19671656 PMC3038121

[B23] Castellano D Sepulveda JM Garcia-Escobar I Rodriguez-Antolin A Sundlov A Cortes-Funes H The role of RANK-ligand inhibition in cancer: the story of denosumab Oncologist 2011 13 2 136 145 10.1634/theoncologist.2010-0154 21285392 PMC3228090

[B24] Saad F Gleason DM Murray R Tchekmedyian S Venner P Lacombe L Chin JL Vinholes JJ Goas JA Chen B A randomized, placebo-controlled trial of zoledronic acid in patients with hormone-refractory metastatic prostate carcinoma J Natl Cancer Inst 2002 13 19 1458 1468 10.1093/jnci/94.19.1458 12359855

[B25] Paller CJ Carducci MA Philips GK Management of bone metastases in refractory prostate cancer–role of denosumab Clin Interv Aging 2012 13 363 372 23049248 10.2147/CIA.S27930 PMC3459574

[B26] Nemeth JA Harb JF Barroso U Jr He Z Grignon DJ Cher ML Severe combined immunodeficient-hu model of human prostate cancer metastasis to human bone Cancer Res 1999 13 8 1987 1993 10213511

[B27] Hsu H Lacey DL Dunstan CR Solovyev I Colombero A Timms E Tan HL Elliott G Kelley MJ Sarosi I Tumor necrosis factor receptor family member RANK mediates osteoclast differentiation and activation induced by osteoprotegerin ligand Proc Natl Acad Sci USA 1999 13 7 3540 3545 10.1073/pnas.96.7.3540 10097072 PMC22329

[B28] Hussein O Tiedemann K Komarova SV Breast cancer cells inhibit spontaneous and bisphosphonate-induced osteoclast apoptosis Bone 2011 13 2 202 211 10.1016/j.bone.2010.09.006 20849994

[B29] Inoue H Nishimura K Oka D Nakai Y Shiba M Tokizane T Arai Y Nakayama M Shimizu K Takaha N Prostate cancer mediates osteoclastogenesis through two different pathways Cancer Lett 2005 13 1 121 128 10.1016/j.canlet.2004.09.053 15890244

[B30] Takayanagi H Kim S Koga T Nishina H Isshiki M Yoshida H Saiura A Isobe M Yokochi T Inoue J Induction and activation of the transcription factor NFATc1 (NFAT2) integrate RANKL signaling in terminal differentiation of osteoclasts Dev Cell 2002 13 6 889 901 10.1016/S1534-5807(02)00369-6 12479813

[B31] Tiedemann K Hussein O Sadvakassova G Guo Y Siegel PM Komarova SV Breast cancer-derived factors stimulate osteoclastogenesis through the Ca2+/protein kinase C and transforming growth factor-beta/MAPK signaling pathways J Biol Chem 2009 13 48 33662 33670 10.1074/jbc.M109.010785 19801662 PMC2785208

[B32] Ishida N Hayashi K Hoshijima M Ogawa T Koga S Miyatake Y Kumegawa M Kimura T Takeya T Large scale gene expression analysis of osteoclastogenesis in vitro and elucidation of NFAT2 as a key regulator J Biol Chem 2002 13 43 41147 41156 10.1074/jbc.M205063200 12171919

[B33] Guo Y Tiedemann K Khalil JA Russo C Siegel PM Komarova SV Osteoclast precursors acquire sensitivity to breast cancer derived factors early in differentiation Bone 2008 13 2 386 393 10.1016/j.bone.2008.03.026 18502714

[B34] Lee SE Woo KM Kim SY Kim HM Kwack K Lee ZH Kim HH The phosphatidylinositol 3-kinase, p38, and extracellular signal-regulated kinase pathways are involved in osteoclast differentiation Bone 2002 13 1 71 77 10.1016/S8756-3282(01)00657-3 11792567

[B35] Chen G Sircar K Aprikian A Potti A Goltzman D Rabbani SA Expression of RANKL/RANK/OPG in primary and metastatic human prostate cancer as markers of disease stage and functional regulation Cancer 2006 13 2 289 298 10.1002/cncr.21978 16752412

[B36] Liu AY Roudier MP True LD Heterogeneity in primary and metastatic prostate cancer as defined by cell surface CD profile Am J Pathol 2004 13 5 1543 1556 10.1016/S0002-9440(10)63412-8 15509525 PMC1618667

[B37] Liu AY Peehl DM Characterization of cultured human prostatic epithelial cells by cluster designation antigen expression Cell Tissue Res 2001 13 3 389 397 10.1007/s004410100419 11572092

[B38] Chen X Rycaj K Liu X Tang DG New insights into prostate cancer stem cells Cell Cycle 2013 13 4 579 586 10.4161/cc.23721 23370446 PMC3594258

[B39] Gordon S Taylor PR Monocyte and macrophage heterogeneity Nat Rev Immunol 2005 13 12 953 964 10.1038/nri1733 16322748

[B40] Ouellet V Tiedemann K Mourskaia A Fong JE Tran-Thanh D Amir E Clemons M Perbal B Komarova SV Siegel PM CCN3 impairs osteoblast and stimulates osteoclast differentiation to favor breast cancer metastasis to bone Am J Pathol 2011 13 5 2377 2388 10.1016/j.ajpath.2011.01.033 21514448 PMC3081179

[B41] Virk MS Petrigliano FA Liu NQ Chatziioannou AF Stout D Kang CO Dougall WC Lieberman JR Influence of simultaneous targeting of the bone morphogenetic protein pathway and RANK/RANKL axis in osteolytic prostate cancer lesion in bone Bone 2009 13 1 160 167 10.1016/j.bone.2008.09.009 18929692 PMC2657045

[B42] Teitelbaum SL The osteoclast and its unique cytoskeleton Ann N Y Acad Sci 2011 13 14 17 10.1111/j.1749-6632.2011.06283.x 22172034

[B43] Chambers TJ Fuller K How are osteoclasts induced to resorb bone? Ann N Y Acad Sci 2011 13 1 6 10.1111/j.1749-6632.2011.06249.x 22172032

[B44] Jin JK Dayyani F Gallick GE Steps in prostate cancer progression that lead to bone metastasis Int J Cancer 2011 13 11 2545 2561 10.1002/ijc.26024 21365645 PMC3082284

[B45] Mishra S Tang Y Wang L deGraffenried L Yeh IT Werner S Troyer D Copland JA Sun LZ Blockade of transforming growth factor-beta (TGFbeta) signaling inhibits osteoblastic tumorigenesis by a novel human prostate cancer cell line Prostate 2011 13 13 1441 1454 10.1002/pros.21361 21321980 PMC3108007

[B46] Barrett JM Rovedo MA Tajuddin AM Jilling T Macoska JA MacDonald J Mangold KA Kaul KL Prostate cancer cells regulate growth and differentiation of bone marrow endothelial cells through TGFbeta and its receptor, TGFbetaRII Prostate 2006 13 6 632 650 10.1002/pros.20370 16388503

[B47] Blanchere M Saunier E Mestayer C Broshuis M Mowszowicz I Alterations of expression and regulation of transforming growth factor beta in human cancer prostate cell lines J Steroid Biochem Mol Biol 2002 13 4–5 297 304 12589936 10.1016/s0960-0760(02)00218-2

[B48] Fuller K Owens JM Jagger CJ Wilson A Moss R Chambers TJ Macrophage colony-stimulating factor stimulates survival and chemotactic behavior in isolated osteoclasts J Exp Med 1993 13 5 1733 1744 10.1084/jem.178.5.1733 8228819 PMC2191238

[B49] Hodge JM Collier FM Pavlos NJ Kirkland MA Nicholson GC M-CSF potently augments RANKL-induced resorption activation in mature human osteoclasts PLoS One 2011 13 6 e21462 10.1371/journal.pone.0021462 21738673 PMC3126821

[B50] Hotokezaka H Sakai E Kanaoka K Saito K Matsuo K Kitaura H Yoshida N Nakayama K U0126 and PD98059, specific inhibitors of MEK, accelerate differentiation of RAW264.7 cells into osteoclast-like cells J Biol Chem 2002 13 49 47366 47372 10.1074/jbc.M208284200 12237315

[B51] He Y Staser K Rhodes SD Liu Y Wu X Park SJ Yuan J Yang X Li X Jiang L Erk1 positively regulates osteoclast differentiation and bone resorptive activity PLoS One 2011 13 9 e24780 10.1371/journal.pone.0024780 21961044 PMC3178550

